# Falls and Injuries After Heading to a Bomb Shelter Following Rocket Warning Sirens in Israel

**DOI:** 10.7759/cureus.109244

**Published:** 2026-05-19

**Authors:** Joseph Palatchi Oldak, Ianiv Trior Simonovich, Galina Shapiro, Yaniv Keren

**Affiliations:** 1 Department of Orthopedic Surgery, Hospital Angeles Universidad, Mexico City, MEX; 2 Department of Orthopedics and Traumatology, Hospital Infantil Privado, Mexico City, MEX; 3 School of Medicine, Universidad Anahuac, Mexico City, MEX; 4 Department of Orthopedics and Traumatology, Rambam Medical Center, Haifa, ISR; 5 Division of Orthopedic Surgery, Rambam Health Care Campus, Haifa, ISR

**Keywords:** falls, fractures, israel, orthopedic injuries, rocket sirens, shelter evacuation

## Abstract

Background: In recent years, Israel has experienced a rise in rocket attacks, forcing civilians to evacuate quickly to bomb shelters. These rapid evacuations have resulted in increased fall-related injuries, particularly among vulnerable groups such as the elderly and those with preexisting medical conditions.

Objective: To assess the patterns of injuries, the contributing factors to falls and orthopedic injuries, particularly fractures, sustained during falls in the process of evacuation to shelters during rocket warning sirens.

Methods: We conducted a retrospective cohort study, including 174 patients treated at Rambam Health Care Campus for falls and injuries sustained during evacuation to bomb shelters following rocket warning sirens.

Results: The mean age was 54.4 years; 106 (60.9%) were female. We found that women were significantly older than men, with a 50% likelihood of being >56 years (*P* = 0.017; relative risk (RR) = 1.50; 95% confidence interval (CI) = 1.07-2.09). The most prevalent injuries were contusions (100, 57.5%) and fractures (60, 34.5%). The most common injury sites were the neck (42, 24.1%), back (29, 16.7%), wrist (23, 13.2%), and knee (21, 12.1%). The predominant chronic diseases were metabolic (74, 42.5%) and cardiovascular (46, 26.4%). Most injuries occurred at home (153, 87.9%).

Conclusion: Falls during shelter evacuation constitute a major orthopedic hazard. Women were significantly older and consequently more susceptible to serious fall incidents. Comorbidities (especially metabolic and cardiovascular) may further increase risk. Preventive measures should focus on house security and the specific protection of high-risk individuals.

## Introduction

During the years 2023 to 2025, Israel experienced a significant increase in rocket warning alerts related to regional security events. These alerts require civilians to rapidly seek shelter within a limited time window, often under conditions of stress, urgency, and reduced situational awareness. This situation has had a profound impact on the physical and mental health of the Israeli population. Fractures and other orthopedic injuries are one of the most commonly observed injury patterns during evacuations [1]. Many falls occur when people rush to shelters. Factors such as overcrowded spaces, the pressure and stress involved, and the rapid nature of the evacuation process can lead to falls and, as a result, fractures. Most susceptible are vulnerable populations such as the elderly, children, and individuals with physical disabilities. Demographic and epidemiological studies of risk factors for falls have been conducted in three broad categories: age, medical, and environmental risk factors [2]. Age-related risk factors include injuries or an age-related decrease in muscle strength, muscle coordination, balance, proprioceptive functions, hearing, and vision [3]. Medical risk factors include the presence of diseases such as diabetes, osteoporosis, and cognitive impairments [4]. Environmental risk factors include poor lighting, wet places, uneven ground and pavements [5], craters, and obstructions in native areas or household places [6]. The most common types of fractures after falling are wrist fractures, ankle fractures, and hip fractures, particularly among older adults [7,8,9]. Despite increasing exposure to such emergency scenarios, there is limited literature evaluating injury patterns and risk factors associated with evacuation-related falls. This study aims to assess the orthopedic injuries, particularly fractures, caused by falls during evacuations to shelters and identify the factors leading to such injuries.

## Materials and methods

This retrospective cohort study was conducted at Rambam Health Care Campus (RHCC), a tertiary referral hospital and primary trauma center in northern Israel. The study period extended from October 2024 to June 2025. A total of 190 patients aged 18-100 years presenting with injuries sustained during evacuation to shelters were identified.

Inclusion criteria comprised adult patients presenting with orthopedic injuries sustained due to a fall while evacuating to a shelter during a warning siren. Patients with injuries directly related to blast exposure, missile impact, or shrapnel were excluded (*n* = 16).

Data were systematically extracted from the institutional electronic medical records system (Prometheus) and imaging archive (PACS) using a standardized protocol. Demographic variables included age and sex. Clinical variables included pre-existing comorbidities categorized as metabolic, cardiovascular, musculoskeletal, pulmonary, or oncologic. Injury-related variables included type of injury (contusion, fracture, sprain, dislocation, or laceration), anatomical location, and injury setting (home, workplace, or public area). Management variables included treatment type, categorized as non-operative or operative, including minimally invasive procedures. Outcome variables included ambulance use and hospital admission.

Continuous variables were reported as mean ± standard deviation (SD), median, and range. Categorical variables were presented as frequencies and percentages. Age was dichotomized at the median (≥56 vs. <56 years). Risk ratios (RR) with 95% confidence intervals (CI) were calculated. Comparisons were performed using chi-square or Fisher’s exact test. A *P*-value < 0.05 was considered statistically significant.

The study was approved by the Institutional Review Board of RHCC (0560-24-RMB-D) and conducted in accordance with the Declaration of Helsinki.

## Results

**Figure 1:**
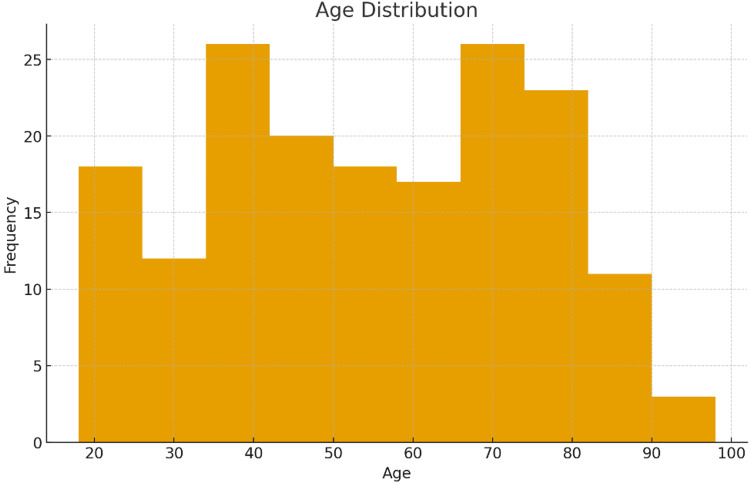
Age distribution. Histogram illustrating the distribution of patient ages (n = 174). Data are presented as frequency counts. The mean age was 54.4 ± 20.3 years, with a median of 56 years. This figure presents descriptive data only; no inferential statistical test was applied.

A total of 174 patients were included in the study. The mean age was 54.4 years (median, 56; SD, 20.3) (Figure 1). A significantly higher proportion of women were in the ≥56 age group compared with men (59.4% vs. 39.7%). Female patients had a 50% increased likelihood of belonging to the older age group (*P* = 0.017; RR = 1.50; 95% CI = 1.07-2.09) (Table 1). The most common injury was contusion (100, 57.5%), followed by fractures (60, 34.5%). Further injuries included lacerations (1, 0.6%), dislocations (2, 1.1%), and sprains (11, 6.3%) (Table 2).

**Table 1:** Association between sex and age group. Data are presented as percentages (%). Risk ratio (RR) with 95% confidence interval (CI) was calculated to assess the association between sex and age group. Female patients demonstrated a higher likelihood of being ≥56 years compared with male patients (RR 1.50; 95% CI 1.07-2.09). Statistical analysis was performed using the chi-square test. A *P*-value of 0.017 was considered statistically significant (*P* < 0.05).

Sex	≥56 years (*n*, %)	RR	95% CI	*P*-value
Female	63 (59.4%)	1.50	1.07-2.09	0.017 (significant)
Male	31 (39.7%)	Reference	Reference	Reference

**Table 2:** Injury characteristics. Data are presented as number (*n*) and percentage (%). This table summarizes the distribution of injury types.

Injury type	Value
Contusion	100 (57.5%)
Fracture	60 (34.5%)
Sprain	11 (6.3%)
Dislocation	2 (1.1%)
Laceration	1 (0.6%)

The majority of injuries occurred inside the home environment, accounting for 153 (88%) of all cases. Although 8 (4.6%) occurred in public spaces, including roads, common areas, or sidewalks, the remaining 13 (7.5%) took place at work.

Table 3 represents the most frequently impacted anatomical area. The neck was the most often affected anatomical area, recorded in 42 (24.1%) of cases, followed by the back, 29 (16.7%), wrist, 23 (13.2%), and knee, 21 (12.1%). Many patients had several medical background conditions. Metabolic diseases were the most prevalent, 74 (42.5%), followed by cardiovascular, 46 (26.4%), musculoskeletal, 7 (4.0%), pulmonary, 2 (1.1%), hepato-renal, 2 (1.1%), other, 2 (1.1%), and malignancy, 1 (0.6%). The remaining 83 (47.7%) had no comorbidities.

**Table 3:** Frequency of injuries by anatomical region. Data are presented as number (*n*) and percentage (%).

Variable	Value
Neck	42 (24.1%)
Back	29 (16.7%)
Wrist	23 (13.2%)
Knee	21 (12.1%)
Pelvis	20 (11.5%)

Most injuries were treated non-operatively, including standard medical management, wound dressing, splinting, and immobilization. Most unstable fractures, particularly those involving the hip, wrist, or pelvis, required further therapeutic intervention. In our study, 30 (17%) underwent surgical treatment, including procedures such as open reduction and internal fixation, external fixation, or arthroplasty. An additional 10 (6%) required minimally invasive procedures, such as percutaneous fixation with pins, screws, or nails, or arthroscopic procedures.

Due to restricted mobility, pain, or suspicion of severe injury at the scene, 101 (50%) required ambulance transport. Hospital admission was necessary in 36 (21%) of the study population.

## Discussion

This study highlights major orthopedic injuries and the repercussions of falls during evacuations to shelters. The high prevalence of long-term comorbidities, including metabolic 74 (42.5%) and cardiovascular 46 (26.4%) disorders, supports the idea that chronic diseases significantly influence the risk of falls, particularly during evacuations. This emphasizes the vulnerability of this population in such situations. These disorders are known to cause neuropathy, orthostatic hypotension, muscle weakness, and reduced proprioception, leading to poor balance, impaired gait, and slower reactions, thereby increasing the likelihood of falls during rapid movements [10].

Fractures accounted for one-third of all fall-related injuries, representing 60 (34%) of the study population. These findings suggest that evacuation-related fractures are not only frequent but also sufficiently severe to require operative management and hospital admission. The high frequency of accidents occurring at home (153, 87.9%) might be due to several reasons, such as the tendency to stay at home during war and crisis or the fact that attacks occurred mostly at times when people were at home.

A review of the Israel Home Front Command alert registry [11] for the city of Haifa during the study period (October 2024 to June 2025) identified a total of 107 rocket- and missile-related warnings. Alerts occurred across all hours of the day; however, they peaked between 07:00-07:59, representing 12 (11.2%) of all events. Secondary peaks were observed during the late afternoon and evening hours, specifically between 16:00-16:59 and 21:00-21:59, each accounting for 9 (8.4%) of events. Early morning periods between 04:00-05:59 also demonstrated elevated alert activity, representing 8 (7.5%) of events.

This highlights the great value of a safe home environment. Narrow corridors, obstructed routes, poor lighting, and slick surfaces during evacuation contributed to injury mechanics. Additionally, psychological stress and panic during warning sirens may contribute to impaired coordination and rushed movements, further increasing fall risk [12]. The high frequency of axial and upper-extremity injuries indicates forward falls and attempts to brace impact, consistent with sudden loss of balance [13]. Although this study was not designed as an interventional or policy-driven investigation, these findings have important implications for public health and emergency preparedness. Targeted interventions such as fall-prevention education, home safety modifications, and personalized evacuation planning for high-risk populations are warranted.

This research is limited by its retrospective design. Comorbidity information was obtained from clinical records and may be underreported. Long-term outcomes, functional rehabilitation, and specific environmental descriptors were not provided.

## Conclusions

Injuries and falls during evacuation to bomb shelters or protected spaces represent a considerable and underrecognized source of harm. In this cohort, older individuals, particularly women, and patients with preexisting comorbidities were more susceptible to fall-related injuries, including a substantial proportion of fractures.

Preventive strategies should focus on improving safety within the home environment, optimizing evacuation routes, and providing targeted education for high-risk populations. Environmental hazards such as poor lighting, obstacles, and confined spaces should be addressed as part of preparedness planning. Although psychological outcomes were not directly assessed in this study, the potential role of psychological support and post-event counseling should be considered within comprehensive emergency preparedness frameworks.

These findings highlight the importance of individualized risk assessment, particularly for vulnerable populations, to optimize evacuation strategies and reduce the risk of injury.
